# Clinical presentation and endoscopic features of primary gastric Burkitt lymphoma in childhood, presenting as a protein-losing enteropathy: a case report

**DOI:** 10.4076/1752-1947-3-7256

**Published:** 2009-06-09

**Authors:** Jenny Hui Chia Chieng, John Garrett, Steven Leslie Ding, Michael Sullivan

**Affiliations:** 1 Department of Paediatrics, Christchurch Hospital, Christchurch, New Zealand; 2 Department of Gastroenterology, Christchurch Hospital, Christchurch, New Zealand; 3 Children's Cancer Research Group, University of Otago, Christchurch, New Zealand

## Abstract

**Introduction:**

Burkitt lymphoma and B cell lymphomas in childhood may arise in many atypical locations, which on rare occasions can include gastric mucosa. A case of primary gastric Burkitt lymphoma is described in a child presenting as a protein-losing enteropathy, including the direct monitoring of the disease response by sequential endoscopic biopsy and molecular analysis.

**Case presentation:**

We report a 9-year-old boy who presented with gross oedema, ascites and respiratory distress caused by a protein-losing enteropathy. Initial imaging investigations were non-diagnostic but gastroduodenal endoscopy revealed massive involvement of the gastric mucosa with a primary Burkitt lymphoma. His subsequent clinical progress and disease response were monitored directly by endoscopy and he remains in clinical remission 4 years after initial diagnosis.

**Conclusions:**

This is the first case report of primary Burkitt lymphoma presenting as a protein-losing enteropathy. The clinical course and progress of the patient were monitored by sequential endoscopic biopsy, histology and molecular analysis by fluorescence in situ hybridisation.

## Introduction

Protein-losing enteropathy (PLE) has many causes including gastrointestinal lymphoma [1-3], however, there are no reports of protein-losing enteropathy caused by a primary gastric lymphoma in childhood. Here we report the clinical presentation, endoscopic features and outcome of a child with PLE caused by Burkitt lymphoma of the stomach.

## Case presentation

A previously healthy 9-year-old boy with normal growth and development presented with progressive pallor, peripheral oedema and respiratory distress. Examination showed pallor, pitting oedema, and respiratory distress. No lymphadenopathy, jaundice, hepatosplenomegaly or abdominal masses were present, and the remainder of the physical examination was normal.

Investigations showed hypoalbuminaemia; albumin 16 g/L, and total protein 27 g/L, with normal liver and renal function. The urine was normal with no proteinuria or haematuria. Haemoglobin (Hb) was 89 g/L, white blood cell count (WCC) 16.6 × 10^9^/L, but neutrophils and lymphocytes and blood film were normal.

The chest X-ray (CXR) showed consolidation in the right lower lobe and an abdominal ultrasound scan was normal.

**Figure 1:**
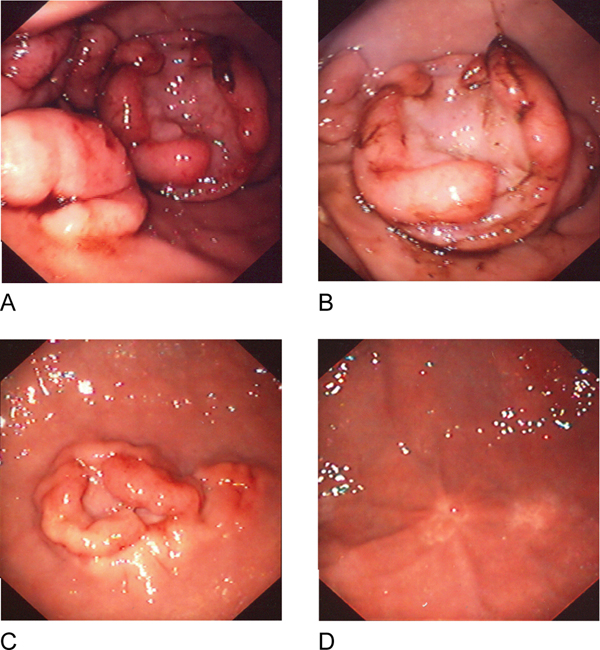
**Gastric endoscopy. (A, B)** Gastric body tumours; multiple large (2 to 3 cm in diameter) raised ulcerated tumours involving the greater curvature of the gastric body and numerous smaller tumours in the second and third part of the duodenum, as far as the gastroscope could reach. **(C)** Gastric body tumour after 1 week of chemotherapy. **(D)** Gastric body tumour after 5 months of chemotherapy demonstrating complete resolution of the lymphomatous masses with only residual scaring present.

At gastroduodenal endoscopy, multiple raised large (2 to 3 cm in diameter) ulcerated tumours of the greater curvature of the gastric body were seen (Figure 1), and numerous smaller tumours were seen in the second and third part of the duodenum but colonoscopy was normal.

**Figure 2:**
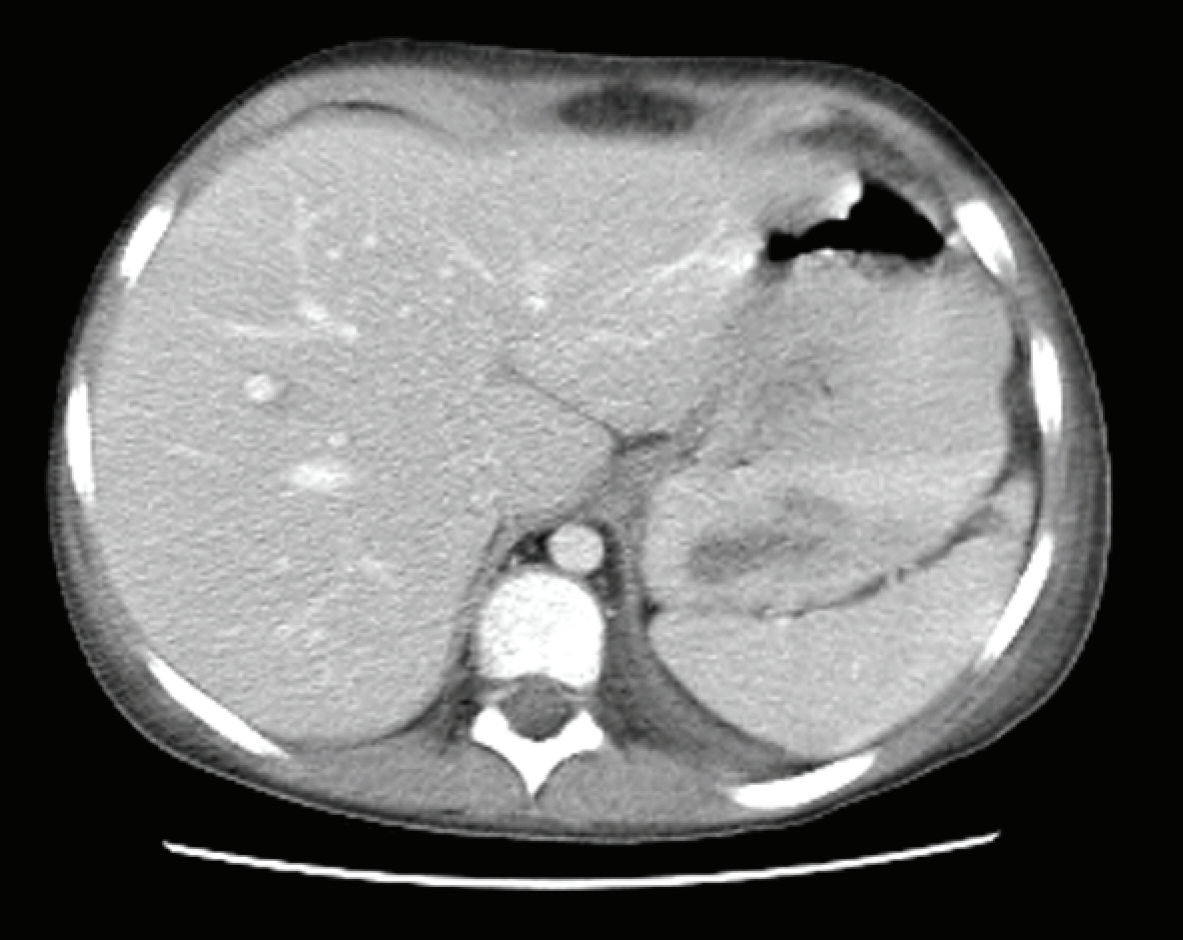
**Computed tomography scan of the abdomen at diagnosis**. Exophytic masses with multiple filling defects are present in the gastric mucosa.

Computed tomography (CT) of the chest and abdomen showed multiple exophytic lesions in the stomach, several filling defects within the jejunum, and a 5 cm long intussusception in the ileum (Figure 2). Small lymph nodes were seen around the superior mesenteric vessels. Bilateral pleural effusions and atelectasis were seen in the chest but there was no mediastinal lymphadenopathy.

**Figure 3:**
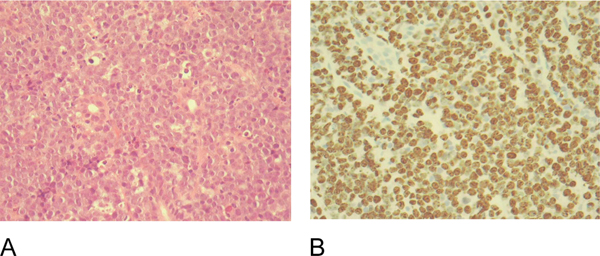
**Histology and immunohistochemistry of gastric biopsies**. **(A)** Haematoxylin and eosin stained section of gastric biopsy showing diffuse small blue round cell infiltrate. **(B)** Immunohistochemistry with CD20 showing gastric infiltrate to be of B-cell lineage.

Gastric biopsies showed a diffuse lymphoid infiltrate (Figure 3A), immunohistochemistry identified a B-cell population and flow cytometry was positive for the B-cell markers CD20, CD10 and CD43 (Figure 3B). Fluorescence in situ hybridisation (FISH) was positive for the C-MYC, t(8:14) translocation of Burkitt lymphoma. The bone marrow aspirate, trephine biopsy and cerebrospinal fluid (CSF) were normal. No *Helicobacter pylori* infection was detected.

The histology, immunophenotype and FISH analysis of the biopsies, and the radiological findings were consistent with a primary gastrointestinal Burkitt lymphoma, Stage III, Group B.

Treatment was according to the UKCCSG consensus guideline for Burkitt lymphoma (2003). Induction chemotherapy with cyclophosphamide, vincristine and prednisone (COP) was given together with alkaline hydration and allopurinol, daily albumin infusions, frusemide and omeprazole.

No tumour lysis syndrome occurred, and there was a rapid rise in serum albumin and protein with resolution of clinical signs of protein-losing enteropathy.

Further chemotherapy included two courses of cyclophosphamide, vincristine, prednisone, cytarabine, doxorubicin and methotrexate (COPADAM) followed by two courses of cytarabine and methotrexate (CYM) and double intrathecal chemotherapy of methotrexate and hydrocortisone.

Our patient's clinical course and disease response were monitored by sequential endoscopic biopsy, histology and molecular analysis by FISH. The rapid clinical response was reflected in the rapid histological and molecular resolution of disease. Follow-up endoscopy showed complete resolution of the mucosal tumours with only residual mucosal puckering present. Abdominal CT cans were normal and, once in remission and having completed chemotherapy, the patient's ongoing disease surveillance was by endoscopy and repeat biopsy for FISH analysis. No molecular evidence of residual disease was detected, and he remains in clinical remission with complete resolution of the protein-losing enteropathy and no treatment related sequelae 4 years from initial diagnosis.

## Discussion

Lymphoma is a well-known cause of protein-losing enteropathy in adults. However, in most cases, it is caused by either diffuse nodal infiltration obstructing intestinal lymphatics or a primary mucosal lymphoma located more distally in the small intestine [1-3]. However, primary gastric lymphoma is rare in the paediatric population, and most of these cases have been associated with gastrointestinal symptoms such as pain, dysphagia, bleeding or gastric outlet obstruction [4-10].

Several cases of primary gastric lymphoma have been described in children in association with concomitant *Helicobacter pylori* infection. Blecker *et al.* described a single case of mucosa-associated lymphoid tissue (MALT) lymphoma more commonly found in adults in a 14-year-old girl with a history of *H. pylori* associated chronic gastritis [11]. More recently, Mezlini *et al.* described two further cases of MALT lymphomas in children with concomitant *H. pylori* infection [12]. These children presented with similar clinical features to those seen in adults.

We report the endoscopic findings of primary gastric Burkitt lymphoma in childhood presenting as a protein-losing enteropathy. The diagnosis here was made difficult by the absence of chronic or acute gastrointestinal symptoms and initial imaging studies also did not indicate a likely aetiology. It was only on endoscopy that other more common causes such as primary intestinal lymphangiectasia, inflammatory diseases and infection were excluded. Here, gastroduodenal endoscopy provided an accurate diagnosis and staging, and was the most useful modality in monitoring the response to treatment.

Burkitt and B-cell lymphomas in childhood have an excellent overall prognosis regardless of the location (except for primary central nervous system (CNS) lymphoma), especially when treated with contemporary chemotherapy protocols [13].

## Conclusion

Primary Burkett lymphoma of the gastric mucosa is uncommon in childhood. We report a child presenting with a protein-losing enteropathy whose subsequent clinical course was monitored by sequential endoscopic biopsy and molecular analysis by FISH. The clinical outcome for Burkitt lymphoma in childhood is excellent, even when presenting in unusual sites with rare clinical manifestations.

## Abbreviations

CNS: central nervous system; COP: cyclophosphamide, vincristine, prednisone; COPADAM: cyclophosphamide, vincristine, prednisone, cytarabine and methotrexate; CSF: cerebrospinal fluid; CT: computed tomography; CXR: chest X-ray; CYM: cytarabine and methotrexate; FISH: fluorescent in situ hybridisation; Hb: haemoglobin; MALT: mucosa-associated lymphoid tissue; WCC: white cell count.

## Consent

Written informed consent was obtained from the patient's parent for publication of this case report and any accompanying images, as the child was a minor. A copy of the written consent is available for review by the Editor-in-Chief of this journal.

## Competing interests

The authors declare that they have no competing interests.

## Authors' contributions

JC and JG abstracted the clinical data and coordinated the initial preparation of images. JC prepared the initial draft manuscript. SD reviewed the endoscopic images and the draft manuscript. MS prepared the images, formatted the figures, and drafted the final manuscript. All authors read and approved the final manuscript.
